# CRISPR/Cas12a-based assay for the rapid and high-sensitivity detection of *Streptococcus agalactiae* colonization in pregnant women with premature rupture of membrane

**DOI:** 10.1186/s12941-023-00558-2

**Published:** 2023-01-19

**Authors:** Donghong Yu, Bin Liang, Haipo Xu, Lu Chen, Zhoujie Ye, Zhihui Wu, Xinrui Wang

**Affiliations:** 1 Medical Research Center, Fujian Maternity and Child Health Hospital, College of Clinical Medicine for Obstetrics and Gynecology and Pediatrics, Fujian Medical University, 350001, Fujian Fuzhou, China; 2 NHC Key Laboratory of Technical Evaluation of Fertility Regulation for Non-human Primate, Fujian Maternity and Child Health Hospital, Fuzhou, Fujian 350013, China; 3 Medical Genetic Diagnosis and Therapy Center, Fujian Key Laboratory for Prenatal Diagnosis and Birth Defect, Fujian Maternity and Child Health Hospital, College of Clinical Medicine for Obstetrics and Gynecology and Pediatrics, Fujian Medical University, Fuzhou, Fujian 350001, China; 4 The United Innovation of Mengchao Hepatobiliary Technology Key Laboratory of Fujian Province, Mengchao Hepatobiliary Hospital of Fujian Medical University, Fuzhou, Fujian 350025 China; 5 College of Chemical Engineering, Fuzhou University, Fuzhou, Fujian 350116 China; 6 Department of Clinical Laboratory, Fujian Maternity and Child Health Hospital, College of Clinical Medicine for Obstetrics and Gynecology and Pediatrics, Fujian Medical University, Fuzhou, Fujian 350001 China

**Keywords:** *Streptococcus agalactiae*, GBS colonization, CRISPR-Cas12a, Recombinase polymerase amplification, Intrapartum screening, Premature rupture of membrane

## Abstract

**Background:**

*Streptococcus agalactiae* or group B *Streptococcus* (GBS) is a leading infectious cause of neonatal morbidity and mortality. It is essential to establish a robust method for the rapid and ultra-sensitive detection of GBS in pregnant women with premature rupture of membrane (PROM).

**Methods:**

This study developed a CRISPR-GBS assay that combined the advantages of the recombinase polymerase amplification (RPA) and CRISPR/Cas12a system for GBS detection. The clinical performance of the CRISPR-GBS assay was assessed using vaginal or cervical swabs that were collected from 179 pregnant women with PROM, compared in parallel to culture-based matrix-assisted laser desorption ionization time-of-flight mass spectrometry (culture-MS) method and real-time quantitative polymerase chain reaction (qPCR) assay.

**Results:**

The CRISPR-GBS assay can be completed within 35 min and the limit of detection was as low as 5 copies μL^−1^. Compared with the culture-MS, the CRISPR-GBS assay demonstrated a sensitivity of 96.64% (144/149, 95% confidence interval [CI] 92.39–98.56%) and a specificity of 100% (30/30, 95% CI  88.65–100%). It also had a high concordance rate of 98.88% with the qPCR assay.

**Conclusions:**

The established CRISPR-GBS platform can detect GBS in a rapid, accurate, easy-to-operate, and cost-efficient manner. It offered a promising tool for the intrapartum screening of GBS colonization.

**Supplementary Information:**

The online version contains supplementary material available at 10.1186/s12941-023-00558-2.

## Background

*Streptococcus agalactiae* or group B *Streptococcus* (GBS) causes 147,000 stillbirths and infant deaths annually worldwide via maternal gastrointestinal and genital tract colonization [1]. Administration of intrapartum antibiotic prophylaxis (IAP) to colonized patients or pregnant women with risk factors has become the primary strategy for the prevention of early-onset disease (EOD) [2, 3]. A key prerequisite for efficient administration of IAP is the reliable antepartum GBS screening [2–4]. Due to the fluctuation of GBS colonization status during pregnancy, accurate rapid GBS diagnostic tests at the time of delivery, would help reduce the use of antibiotic prophylaxis in women who are not colonized and would further reduce EOD cases [5–7].

The current CDC guidelines advocated a universal culture-based screening for GBS colonization during the 35–37th gestational weeks [2]. Nevertheless, the identification results of culture methods are not available until 48 h later, making it unsuitable for pregnant carriers who deliver precipitately [8]. Furthermore, approximately 5–8% of non-haemolytic and/or non-pigmented GBS strains lead to a false-negative result, suggesting the inadequate sensitivity of culture-based screening tests [9]. Several Food and Drug Administration-cleared nucleic acid amplification tests (NAATs), such as BD MAX system (Becton, Dickinson) and the *Illumigene* system (Meridian Bioscience) have been proved to shorten the time-to-result and improve the detection sensitivity [4, 10]. One limitation is the requirement of specialized instruments to provide the heat and cool process that has largely bound their deployment in under-resourced areas. Therefore, the demand for instrument-free nucleic acid detection technologies has driven the development of isothermal amplification methods, such as real-time recombinase polymerase amplification (RT-RPA) [11] and real-time fluorescence loop-mediated isothermal amplification (RT-LAMP) assays [12]. These methods do not rely on thermal cycling but are considerably less sensitive than real-time quantitative polymerase chain reaction (qPCR)-based methods [11–15]. Consequently, a rapid, accurate, and easy-to-implement method is required to facilitate the intrapartum screening of GBS.

Recently, clustered regularly interspaced short palindromic repeat (CRISPR)-associated proteins (Cas) systems have shown to be advantageous for portable detection of pathogenic and nonpathogenic nucleic acids [16, 17]. These approaches rely on the Cas proteins, which can be activated to cleave single-stranded DNA (ssDNA) or RNA nonspecifically after binding to a specific target via the programmable CRISPR RNA (crRNA) [18, 19]. In combination with nucleic acid pre-amplification techniques, such as PCR, RPA, and LAMP, CRISPR/Cas systems exhibit extremely high sensitivity (zettamolar) and specificities of 1–2 nt [20–23].

In this study, we developed a CRISPR/Cas12a-based tool for the detection of GBS, namely the CRISPR-GBS assay. The analytical and clinical performances of the CRISPR-GBS assay were systematically evaluated comparing to culture-based matrix-assisted laser desorption ionization time-of-flight mass spectrometry (culture-MS) and qPCR assay. For clinical evaluation, a retrospective, comparative study was performed using vaginal or cervical swabs collected from 179 pregnant women with premature rupture of membrane (PROM).

## Methods

### Oligonucleotide design and synthesis

The DNA sequence of the *cfb* gene (GenBank accession no. X72754.1) was retrieved from the NCBI website (http://www.ncbi.nlm.nih.gov) and cloned into the pUC57 vector to generate the GBS plasmid pUC57-*cfb.* RPA primers were designed specifically for the *cfb* gene. The ssDNA reporter labeling with FAM and BHQ-1 was used for the Cas12a/crRNA reaction. For qPCR assay, the forward and reverse primers as well as the probe targeting the *cfb* gene as previously described [13], were used in this study. All primers, DNA/RNA oligonucleotides, and probes were synthesized by Sangon Biotech (Shanghai, China), and the sequences information were listed in Additional file 2: Table S1.

### Design and preparation of crRNAs

The crRNAs were designed according to the target sequence of *cfb*, and their target efficiency was scored using the CRISPR-DT online software (http://bioinfolab.miamioh.edu/CRISPR-DT/interface/Cpf1_efficiency.php) for Cpf1 [24]. The secondary structure and the minimum free energy (MFE) of these crRNAs were further evaluated using NUPACK (http://www.nupack.org/). Then, the homology of the crRNAs was analyzed using Nucleotide BLAST (https://blast.ncbi.nlm.nih.gov/Blast.cgi). The selected crRNAs were synthesized by Sangon Biotech (Shanghai, China). The crRNA that exhibited the highest efficiency was used in the subsequent detection assay.

### CRISPR/Cas12a fluorescence assay

RPA was performed using the TwistAmp^™^ Basic kit (TwistDx, Cambridge, UK). Briefly, 12.5 μL of the total reaction volume contained the following: 1 × rehydration buffer, 480 nmol L^−1^ of both forward and reverse primers, 2 μL diethylpyrocarbonate water, 2.5 μL of the DNA template and 1 μL of magnesium acetate (MgOAc; final concentration: 14 mmol L^−1^). The reaction tubes were incubated at 37 °C for 15 min, including a manual mixing step (5-s tube vortex) in the fourth minute. For the no-template control (NTC), these reactions were prepared by substituting the DNA template with an equal volume of molecular grade water.

After RPA, 12.5 μL of RPA products were mixed with 7.5 μL of Cas12a reaction mixture, which contained 100 nmol L^−1^ of crRNA, 50 nmol L^−1^ of ssDNA reporter, 50 nmol L^−1^ of LbaCas12a (EnGen LbaCas12a, M0653T, NEB), 1 × NEBuffer 2.0 (New England Biolabs, UK), and 1.5 μL of RNase-free water, for a final volume of 20 μL. Then, the reaction was incubated at 37 °C using the Applied Biosystems ^™^ Quant Studio 3 (Thermo Fisher Scientific, USA) and fluorescence was measured every minute.

### Analytical studies of CRISPR-GBS assay

For the sensitivity assay of the CRISPR-GBS fluorescence detection, we evaluated the limit of detection (LoD, i.e., minimal number of copies that can be detected) using the GBS genomic DNA (gDNA). The reference strain of GBS (ATCC 12,386) was obtained from the Department of Microbiology Laboratory, Fujian Maternity and Child Health Hospital (Fuzhou, China). Template stock concentrations were analyzed using the NanoDrop^™^ One spectrophotometer (Thermo Fisher Scientific, USA) prior to dilution. GBS gDNA (2.07 Mb) was obtained from the NCBI Reference Sequence (GenBank accession no. NZ_CP012480). DNAs were serially diluted in RNase-free water to 10^5^, 10^4^, 10^3^, 10^2^, 10^1^, 5, and 1 copy μL^−1^, respectively. Then, 2.5 μL of each diluted solution was added to the RPA mixture for amplification. Finally, 12.5 μL of the RPA product was transferred into the Cas12a reaction mixture. Eight replicates were performed per concentration, and the LoD was determined by statistical significance of the lowest copy number experimental group compared to the NTC.

For the specificity assay, *Streptococcus agalactiae* (ATCC 12386) and other sixteen microbial species were obtained from the Department of Microbiology Laboratory, Fujian Maternity and Child Health Hospital (Fuzhou, China). The microbial species were as follows: *Trichomonas vaginalis*, *Streptococcus pyogenes*, *Candida albicans*, *Ureaplasma urealyticum*, *Gardnerella vaginalis*, *Bifidobacterium breve*, *Neisseria gonorrhoeae*, *Lactococcus lactis*, *Escherichia coli*, *Acinetobacter baumannii*, *Streptococcus pneumoniae*, *Staphylococcus aureus*, *Pseudomonas aeruginosa*, *Bacteroides fragilis*, *Enterococcus faecalis* and *Lactobacillus crispatus*. All strains were suspended in 1 mL of TE buffer (10 mmol L^−1^ Tris–HCl, pH8.5, 1 mmol L^−1^ EDTA, and 1% TritonX-100). The gDNA was extracted using the heating lysis method at 100 °C for 10 min, and the supernatants were collected via centrifugation at 12,000 × *g* for 1 min. The concentration of the gDNA was quantified using the NanoDrop^™^ one spectrophotometer and diluted to 1 × 10^5^ copies μL^−1^ for use. Then, 2.5 μL of each DNA template was added to the reaction mixtures for RPA. Three replicates were performed at each data point.

### Culture-MS method identification of GBS

Vaginal or cervical swabs were inoculated onto column blood agar (Bioivd, China) at 37 °C in 5% CO_2_ for 18–24 h. Negative plates were incubated for an additional 24 h prior to signing out. Then, the presumptive, beta-hemolytic GBS colonies were selected to undergo a confirmatory test using matrix-assisted laser desorption ionization time-of-flight mass spectrometry (Bruker Biotyper, BD). The culture-MS identification was completed by medical workers in the Department of Microbiology Laboratory of Fujian Maternity and Child Health Hospital.

### Clinical study of CRISPR-GBS assay

The clinical performance of CRISPR-GBS assay was evaluated parallel to qPCR and culture-MS. A total of 179 vaginal or cervical swab specimens from pregnant women with PROM were collected from 2020 to 2021 at the Fujian Maternity and Child Health Hospital (Fuzhou, China). All specimens were obtained in duplicate from each participant, one for culture-MS assay performed in the Department of Microbiology Laboratory, and the other for DNA extraction via heating lysis as described above. The supernatant was used as the gDNA for the CRISPR-GBS and qPCR assay, which performed on the Applied Biosystems ^™^ Quant Studio 3.

### Determination of cut-off value for the CRISPR-GBS assay

For analysis of the clinical performance of the CRISPR-GBS assay, a total of 30 clinical samples (15 negative and 15 positive samples tested by the culture-MS method) were randomly selected to analyze using the receiver operating characteristic (ROC) [25, 26]. The cut-off value was determined using the maximum Youden index, calculated using the following formula: Youden index = sensitivity + specificity−1. ROC was statistically analyzed using the GraphPad Prism software (version 5.0), while the Youden index was determined using Microsoft Excel (2016).

### Conventional qPCR assay for GBS detection

Conventional qPCR was used to validate the CRISPR-GBS assay. The reaction with a total volume of 25 μL, contained 1U of *Taq* DNA polymerase (Hot Start Version; Takara Bio, Japan), 3.75 mmol L^−1^ of MgCl_2_, 0.25 mmol L^−1^ of dNTP solution mix, 2.5 μmol L^−1^ of TaqMan probe, 0.2 μmol L^−1^ of the forward primer, 0.2 μmol L^−1^ of the reverse primer, 11 μL of PCR-grade water, 1 × PCR buffer (Takara Bio), and 5 μL of the DNA. A touchdown qPCR method was performed as follows: a denaturation step at 95 °C for 10 min, a touchdown program was performed with 10 cycles at 95 °C for 30 s and 65 °C for 60 s (− 1 ℃ per cycle), followed by 40 cycles at 95 °C for 30 s and 55 °C for 60 s. Fluorescence was collected at 55 °C. This process was performed using the Applied Biosystems ™ Quant Studio 3. Optimal C_T_ cut-off value was determined by calculating the Youden Index of ROC curve as described above [27].

### Statistical analysis

All results generated from at least three technical replicates were presented as the mean ± standard deviation (SD) and compared using Dunnett’s multiple comparisons test. Statistical significance was set at *P* < 0.05. Statistical analyses and figures were conducted and generated, respectively, using IBM SPASS Statistics 23, Origin Lab version 8.0, GraphPad Prism 5.0, and Adobe Illustrator CS5. The sensitivity, specificity, kappa value (κ), overall agreement percentage (OPA), positive percentage agreement (PPA), and negative percentage agreement (NPA), in agreement with two-sided 95% confidence intervals (CIs), were analyzed using OpenEpi (http://wwww.openepi.com/Menu/OE_Menu.htm).

## Results

### Design of the CRISPR-GBS assay

**Fig. 1:**
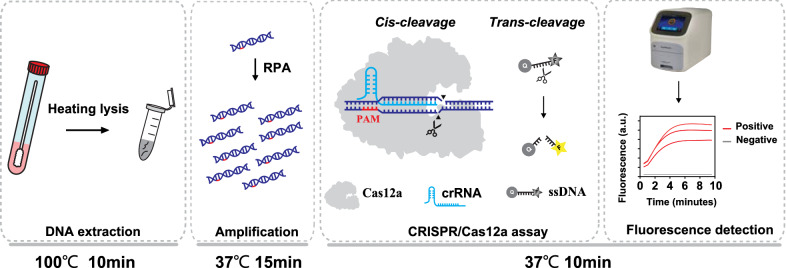
Schematic diagram of the CRISPR-GBS assay. Genomic DNA was extracted and the target gene was amplified by RPA. Positive fluorescence signals were produced when ssDNA probes were collateral cleaved by activated Cas12a after crRNA recognized the PAM sequence of target gene. *RPA* recombinase polymerase amplification, *PAM* protospacer-adjacent motif, *ssDNA* single-stranded DNA

We developed a rapid, highly-sensitive, and easy-to-implement GBS detection assay by combining RPA reaction with the CRISPR/Cas12a step, as illustrated in Fig. 1. To simplify operation to achieve on-site detection pattern, gDNA from vaginal or cervical swabs were extracted crudely via heating lysis. A highly conserved target of the *cfb* gene, which encodes the Christie-Atkins-Munch-Petersen (CAMP) factor, was selected to be amplified using RPA. Then, the crRNA-directed binding *cfb* target activated the *cis*-cleavage activity of Cas12a, followed by the *trans*-cleavage of ssDNA, yielding a fluorescence signal. Within a short duration, the GBS-positive samples exhibited strong fluorescence signals compared to those in negative samples.

**Fig. 2:**
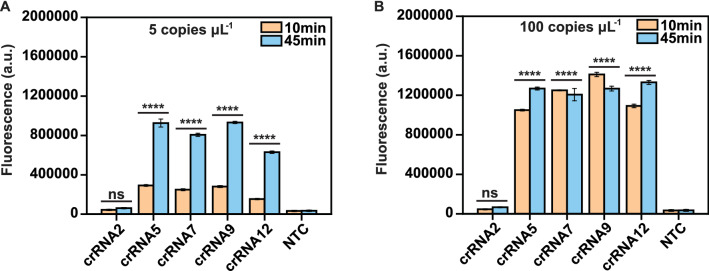
Screening for highly active crRNA. Five crRNAs were evaluated using DNA concentrations of 5 copies μL^−1^ **A** and 100 copies μL^−1^ **B** for 10 min (orange color) and 45 min (blue color). Dunnett’s multiple comparisons test was used to analyze the difference from NTC. Error bars represent mean ± SD, n = 3 technical replicates; ∗∗∗∗ *P* ≤ 0.0001; ns, not significant; NTC, no template control; A.U., arbitrary units

With the goal of robust Cas12a-based recognition and species-level discrimination of GBS, multiple crRNAs were designed according to a 5′-TTTN-3′ protospacer-adjacent motif (PAM) of the target amplification sequences, and then scored using the Cpf1-CRISPR-DT online software. Ultimately, twelve crRNAs with target efficiency scores > 0.3 were included (Additional file 2: Table S2). Meanwhile, according to previous study, the crRNA with the correct hairpin structure in its backbone sequence [28] and lower MFE was more efficient than others [29]. Therefore, the secondary structure of the twelve crRNAs and corresponding MFE values were predicated using the NUPACK online tools, as shown in Additional file 1: Fig.S1. Afterwards, five crRNAs (crRNA2, crRNA5, crRNA7, crRNA9, and crRNA12) were selected to evaluate the efficacy by measuring the total fluorescent signal produced in the presence of two different concentrations of target (5 copies μL^−1^ and 100 copies μL^−1^) over 10 min and 45 min (Fig. 2A, B). Consequently, considering the total intensity of signal and the rate of activation, crRNA9 showed the high performance and was selected for the remaining evaluations.

### Optimization of the CRISPR-GBS assay

**Fig. 3:**
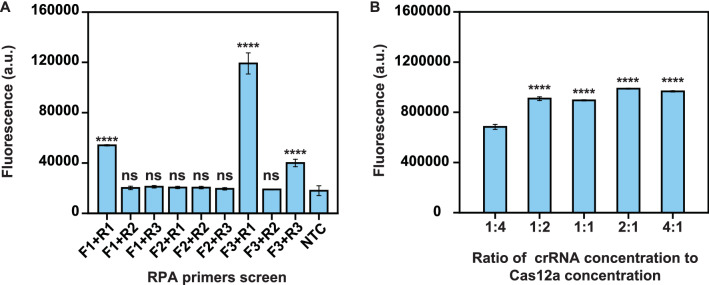
Establishing the CRISPR-GBS fluorescence assay. **A** Determination of the optimal primer sets for CRISPR-GBS fluorescence assay using 100 copies μL^−1^ DNA template. The fluorescence signals were obtained at 10 min. Dunnett’s multiple comparisons test was used to analyze the difference from NTC. **B** Optimizing the concentration ratio of crRNA to LbCas12a. Dunnett’s multiple comparisons test was used to analyze the difference from the ratio of 1:4. Error bars in **A**, **B** represent the mean ± SD, n = 3 replicates

RPA, an enrichment step of target gene, is crucial to provide sufficient substrates for crRNA binding and subsequent activation of the Cas12a protein. To screen out the best primer set, nine RPA primer combinations targeting the *cfb* gene were designed; the sequence information are summarized in Additional file 2: Table S1. Prominently, the primer set of F3 + R1 exhibited the highest amplification efficiency and was adopted for CRISPR-GBS assay (Fig. 3A). The expected product length for this primers set was 212 bp.

To further optimize our assay, we also investigated the appropriate concentration ratio of crRNA to LbCas12a. The results demonstrated that 2:1 ratio performed better than others (Fig. 3B). Moreover, considering our whole CRISPR-GBS assay workflow suitable for field-deployable diagnostics, we adopted a reaction temperature of 37 °C.

### Sensitivity analysis of the CRISPR-GBS assay

**Fig. 4:**
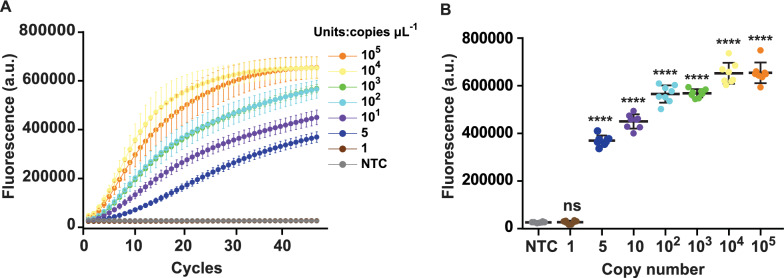
Sensitivity analysis of the CRISPR-GBS assay for GBS detection. **A** Representative plot of fluorescence intensity versus time for Cas12a reaction at 45 min. **B** Endpoint fluorescence signal was obtained at 45 min. Data represents mean ± SD from octuplicate measurements. Dunnett’s multiple comparisons test was used to analyze the difference from NTC

For sensitivity analysis of the CRISPR-GBS fluorescence assay, the LoD was evaluated using serially diluted GBS gDNA. Consequently, we observed a significant increase fluorescence signal for concentrations ≥ 5 copies μL^−1^; it was more prominent in higher copy groups (Fig. 4A, B). The LoD for CRISPR-GBS detection was 5 copies μL^−1^, indicating an attomolar analytical sensitivity comparable to other CRISPR systems [23, 24, 30]. Interestingly, we also observed that at the tenth minute, the positive signal of DNA concentrations ≥ 5 copies μL^−1^ could be clearly distinguished from the negative one. This indicated that the detection time of the CRISPR/Cas12a assay could be shortened to 10 min (Additional file 1: Fig. S2).

### Specificity analysis of the CRISPR-GBS assay

**Fig. 5:**
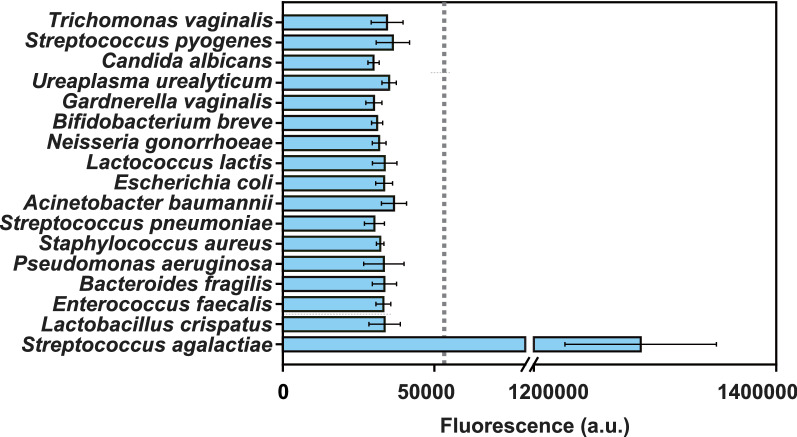
Specificity analysis of the CRISPR-GBS assay for GBS detection. The dashed line means the cut-off value of CRISPR-GBS assay determined by the ROC curve

A highly specific detection method can avoid cross-reactions and improve the accuracy of detection. To assess the specificity of the CRISPR-GBS assay, sixteen microbial species, including several gastrointestinal and vaginal microorganisms, were tested. Consequently, according to the judgment of criteria above, no positive results were obtained from the tested gDNA, except for the GBS strains, which demonstrated that CRISPR-GBS assay had a high specificity for GBS detection (Fig. 5).

### Clinical evaluation of CRISPR-GBS assay

**Fig. 6:**
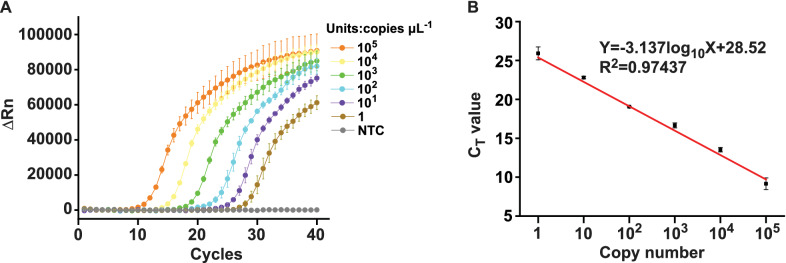
Establishment of a qPCR assay as a comparative method for GBS detection. **A** The performance of qPCR detection method was validated by DNA concentrations in the range of 1 to 10^5^ copies μL^−1^. **B** A standard curve of qPCR assay for quantification of GBS. C_T_ values were plotted against copy numbers of GBS plasmid DNA. Data represents mean ± SD from triplicate measurements. ∆Rn = Rnf-Rnb, where Rnf was the fluorescence emission of the product at each time point and Rnb was the fluorescence emission of the baseline

A total of 179 vaginal or cervical swab specimens from pregnant women with PROM was collected to evaluate the clinical performance of the CRISPR-GBS assay using culture-MS as the reference method. The cut-off value of the CRISPR-GBS assay was determined by the ROC curve plotting (Additional file 1: Fig. S3A), which was calculated to be 53,259 (a.u.); that is, fluorescence signal values higher than 53,259 (a.u.) were considered positive, while lower were negative. Meanwhile, a qPCR assay was established in parallel as a molecular comparative method with a C_T_ cutoff value of 25.89 (Additional file 1: Fig. S3B). The LoD of our qPCR method was 1 copy μL^−1^ (Fig. 6), equivalently to previous study [13].

**Fig. 7:**
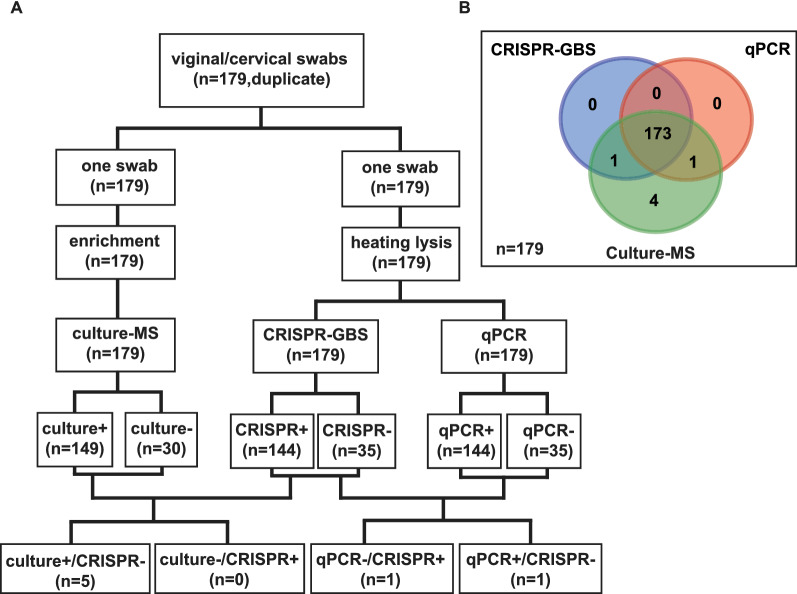
The clinical performance of CRISPR-GBS assay. **A** Study design of CRISPR-GBS assay and results summary as categorized by culture-MS, CRISPR-GBS and qPCR. + , positive results; - , negative results. **B** Venn diagram shows the results of GBS detection by CRISPR-GBS (blue circle), qPCR (red circle) and culture-MS (green circle) assays using clinical samples (n = 179)

Of the 179 specimens, the CRISPR-GBS assay identified 144 GBS-positive samples out of 149 culture-MS positive specimens and correctly detected 30 culture-MS negative specimens (Table 1, Fig. 7A and B). Among the five samples undetected by CRISPR-GBS assay, four samples (nos. 52, 62, 63, and 85) were also undetectable by qPCR. Only one sample (no. 42) was GBS-positive by qPCR, with a C_T_ value of 23.11, indicating lower copies of gDNA (Table 2). Overall, our result showed a clinical sensitivity of 96.64% (144/149, 95% CI  92.39–98.56%) and a clinical specificity of 100% (30/30, 95% CI  88.65–100%). A high kappa value (κ) of 0.9061 (*P* < 0.001) indicated a good correlation between the CRISPR-GBS assay and the culture-MS method (Table 1).

**Table 1:** Performance of CRISPR-GBS assay in clinical samples compared with culture-MS method

CRISPR-GBS assay	Culture-MS method			Comparison of two methods		
	Positive	Negative	Total	%Sensitivity	%Specificity	Kappa
Positive	144	0	144	96.64 (92.39–98.56)^*^	100 (88.65–100)	0.9061 (0.7603–1.052)
Negative	5	30	35			
Total	149	30	179			

*CRISPR-GBS* CRISPR-based method for GBS detection,* Culture-MS* culture method combined with matrix-assisted laser desorption/ionization time-off light mass spectrometry

^*^Values in parentheses are the 95% CI, two-sided 95% confidence interval

**Table 2:** Details of the six discordant samples among CRISPR-GBS, qPCR, and culture-MS method

Sample no	Source	Individual test result		
		CRISPR-GBS	qPCR^b^ (C_T_ value)	Culture-MS result
42	cervix	Negative	Positive (23.11)	Positive
52	cervix	Negative	Undetected (0)	Positive
62	cervix	Negative	Undetected (0)	Positive
63	cervix	Negative	Negative (32.64)	Positive
75	cervix	784,806.94^a^	Negative (31.79)	Positive
85	cervix	Negative	Negative (26.59)	Positive

^a^Fluorescence signal value (a.u)

^b^qPCR result was judged as positive when the cycle threshold (C_T_) value was > 0 and ≤ 25.89

Comparing the CRISPR-GBS with qPCR assay, two discordant results were observed (nos. 42 and 75). In contrast to sample no. 42, sample no. 75 was identified as GBS-positive by the CRISPR-GBS but was undetected by qPCR assay (Table 2). Overall, the results demonstrated that CRISPR-GBS was highly concordant with the qPCR assay, with OPA of 98.88%, PPA of 99.31%, and NPA of 97.14% (Table 3).

**Table 3:** Comparison of the clinical performance of the CRISPR-GBS with qPCR assay

	CRISPR-GBS			Comparison of two methods		
qPCR	Positive	Negative	Total	%OPA^a^	%PPA^b^	%NPA^c^
Positive	143	1	144	98.88 (96.02–99.69)^d^	99.31 (96.17–99.88)	97.14 (85.47–99.49)
Negative	1	34	35			
Total	144	35	179			

^a^OPA, Overall percent agreement

^b^PPA, Positive percent agreement

^c^NPA, Negative percent agreement

^d^Values in parentheses are the 95% CI, two-sided 95% confidence interval

## Discussion

Despite the substantial reductions in the burden of EOD, continued efforts to develop universal screening tests for GBS remains to be the cornerstones of neonatal disease prevention [2]. It is a challenging task to establish a sufficiently sensitive test using nonenriched specimens to detect GBS colonization reliably in the intrapartum setting [2]. In this study, we developed a CRISPR-GBS assay for GBS detection by combining the advantages of isothermal amplification and the high-sensitivity of Cas12a/crRNA *trans*-cleavage. The analytical sensitivity study indicated that the CRISPR-GBS assay could detect as low as 5 copies μL^−1^ of gDNA, which is comparable to the qPCR assay (two to three genomic copies) [13], but is more sensitive than the RT-RPA assay (98 genome copies per reaction) [11]. Clinical evaluation using 179 specimens showed good performance compared with the culture-MS method and qPCR assay.

The CRISPR-GBS assay mainly distinguishes itself from the current systems in terms of its potential application for intrapartum screening. As for experimental conditions, the incubation temperatures used in the entire protocol (100℃ for heating lysis and 37 ℃ for RPA and Cas12a/crRNA reaction) can be achieved using any constant temperature incubator, and the final readout can be obtained, in the best of cases with a real-time thermocycler, but also with the naked eye [31–34]. Additionally, in urgent situations where reports needed to be sent to clinicians immediately (e.g., PROM), the CRISPR-GBS assay has the prominent advantage in terms of speed. The total time was within 35 min, including 10 min for heating lysis, 15 min for the RPA and 10 min for the Cas12a/crRNA analysis. Notably, nonenriched specimens processed directly via heating lysis in closed-tube manner instead of time-consuming extraction steps can not only decrease the turnround time, but also effectively avoid the risk of aerosol contamination. Moreover, the labor costs (equipment and personal were not included) for the CRISPR-GBS were estimated to be only $0.613 [23, 24], which was cost-effective compared to that of culture screening ($4.95 *per* swab) [35] and qPCR-based detection ($7 *per* birth) [36, 37]. A systematic comparison between the current GBS diagnostic assays is provided in Table 4. Together, these features demonstrated that CRISPR-GBS can be further optimized to be an on-site, point-of-care testing for the intrapartum screening of GBS.

**Table 4:** Comparison of the CRISPR-GBS assay with several current GBS detection methods

	RT-PCR [13]	RT-LAMP [12]	RT-RPA [11]	CRISPR-GBS
LoD	2–3 genome copies	300 pg μL^−1^	98 genomic copies	5 copies μL^−1^
Cross-reaction	No	No	No	No
Turnaround time	Detection time of 35 min (exclude DNA extraction)	Detection time of 60 min (exclude DNA extraction)	Detection time of 30 min (exclude DNA extraction)	Turnaround time of 35 min (include DNA extraction)
DNA extraction	DNA extraction kit (G NOME kit)	DNA extraction kit	DNA extraction kit (BD GeneOhm^™^ Strep B kit)	Heating lysis
Target	*cfb* gene	*fbs* gene	*cfb* gene	*cfb* gene
Temperature	Temperature cycling	A constant temperature of 63 ℃	A constant temperature of 39 ℃	A constant temperature of 37 ℃
Clinical samples	15 vaginal swabs	None	50 vaginal/anal samples	179 vaginal/ cervical swabs
Cost	Unknown	Unknown	Around $ 10	Around $ 0.6138 [23, 24]

The clinical evaluation of CRISPR-GBS assay using 179 samples from pregnant women with PROM showed the sensitivity of 96.64% and the specificity of 100% compared to the culture-MS method; thus highlights its potential for GBS identification. The reference method of culture-MS has been confirmed to be more sensitive and accurate than the conventional culture for GBS identification [38, 39]. Moreover, CRISPR-GBS demonstrated a high concordance of 98.88% with the qPCR assay. A total of six discordant samples between culture-MS and the two molecular assays, CRISPR-GBS and qPCR, were observed (Table 2). These discrepancies may be due to the use of different swabs obtained from each participant, which may have heterogeneous bacterial loads, especially for individuals with low bacterial loads. Of note, all these six discordant samples were collected from the cervix, which could lead to a substantial reduction of coccus per swab compared to those from both the lower vagina and rectum swabs [2, 40].

Due to the stringent criteria required for diagnostics, further optimization in the advancement of quantification, workflow, and deployment are necessary to develop CRISPR-GBS to its full potential. One limit of this study is the difficulty in quantifying the true GBS bacterial load in clinical samples. The DNA extraction was subjected to RPA prior to CRISPR/Cas12a reaction, which rapidly reached a signal plateau owing to its high efficiency. Additionally, DNA templates were prepared via crude extraction, that is, heating lysis in this study; thus, it is unclear whether CRISPR-GBS and qPCR were blocked by some inhibitors. To resolve this, a third swab should be collected and processed using the standard DNA extraction/purification methods for both CRISPR-GBS and qPCR assays in comparison with crude lysates. Moreover, CRISPR-based duplex detection system containing the internal amplification control (e.g., *RNase P*) to verify the efficiency should be explored in future work.

## Conclusions

This study successfully developed a novel CRISPR-GBS test for the timely detection of GBS colonization in pregnant women with PROM. This assay is rapid, portable, and cost-efficient that could be recommended as an alternative tool for GBS intrapartum screening. It allows clinicians to determine the most suitable options for IAP during delivery.

## Supplementary Information

**Additional file 1. Figure S1.** Predicted the second structure and minimum free energy of crRNA1 to crRNA12. *Abbreviation: *MFE, the minimum free energy. **Figure S2. **Sensitivity analysis of the CRISPR-GBS assay for GBS detection. Endpoint fluorescence signals of Cas12a reaction were obtained at 10min. Data represents mean ± SD from octuplicate measurements. Dunnett’s multiple comparisons test was used to analyze the difference from NTC (10^5^, 10^4^, 10^3^, 10^2^, 10^1^ vs NTC). ****P ≤ 0.0001; **P ≤0.05. ns, not significant; NTC, no template control; A.U., arbitrary unit. **Figure S3.** ROC curve analysis of the CRISPR-GBS assay and qPCR assay on clinical samples. (A) The ROC analysis of the performance of CRISPR-GBS assay on clinical samples. The cut-off value of CRISPR-GBS assay was determined using the maximum Youden index, which was calculated using the following formula: Youden index = sensitivity + specificity – 1. The ROC curve was statistically analyzed using the GraphPad Prism software (version 5.0), and the Youden Index was determined using Microsoft Excel (2016). (B) The ROC analysis of the qPCR assay on clinical samples. The cut-off value of C_T_ was determined using the maximum Youden index. AUC, Area Under Curve.

**Additional file 2. Table S1. **The oligonucleotide sequences of plasmid, primers and probe used in this study. **Table S2. **The sequences and the corresponding target efficiency score of designed crRNAs.

## Data Availability

The data that support the findings of this study are openly available in figshare.2014. (https://doi.org/10.6084/m9.figshare.17149208.v12).

## Abbreviations

CAMP: Christie-Atkins-Munch-Petersen
Cas: CRISPR-associated protein
CDC: Centers for Disease Control and Prevention
CI: Confidence interval
CRISPR: Clustered regularly interspaced short palindromic repeats
crRNA: CRISPR RNA
CT: Cycle threshold
culture-MS: Culture-based matrix-assisted laser desorption ionization time-of-flight mass spectrometry
EOD: Early-onset disease
GBS: Group B Streptococcus
gDNA: Genomic DNA
IAP: Intrapartum antibiotic prophylaxis
κ: Kappa
LOD: Limit of detection
MFE: Minimum free energy
NAATs: Nucleic acid amplification tests
NPA: Negative percentage agreement
NTC: No-template control
OPA: Overall agreement percentage
PAM: Protospacer-adjacent motif
PROM: Premature rupture of membrane
PPA: Positive percentage agreement
qPCR: Real-time quantitative polymerase chain reaction
ROC: Receiver operating characteristic
RT-RPA: Real-time recombinase polymerase amplification
RT-LAMP: Real-time fluorescence loop-mediated isothermal amplification
SD: Standard deviation
ssDNA: Single-stranded DNA
