# Supplementary material for: CRISPR/Cas12a-based assay for the rapid and high-sensitivity detection of Streptococcus agalactiae colonization in pregnant women with premature rupture of membrane
Source: Ann Clin Microbiol Antimicrob. 2023 Jan 19;22:8. doi: 10.1186/s12941-023-00558-2 (PMC9854146; doi:10.1186/s12941-023-00558-2)
Supplement: Supplementary file 1 — Additional file 1. Figure S1. Predicted the second structure and minimum free energy of crRNA1 to crRNA12. Abbreviation: MFE, the minimum free energy. Figure S2. Sensitivity analysis of the CRISPR-GBS assay for GBS detection. Endpoint fluorescence signals of Cas12a reaction were obtained at 10min. Data represents mean ± SD from octuplicate measurements. Dunnett’s multiple comparisons test was used to analyze the difference from NTC (105, 104, 103, 102, 101 vs NTC). ****P ≤ 0.0001; **P ≤0.05. ns, not significant; NTC, no template control; A.U., arbitrary unit. Figure S3. ROC curve analysis of the CRISPR-GBS assay and qPCR assay on clinical samples. (A) The ROC analysis of the performance of CRISPR-GBS assay on clinical samples. The cut-off value of CRISPR-GBS assay was determined using the maximum Youden index, which was calculated using the following formula: Youden index = sensitivity + specificity – 1. The ROC curve was statistically analyzed using the GraphPad Prism software (version 5.0), and the Youden Index was determined using Microsoft Excel (2016). (B) The ROC analysis of the qPCR assay on clinical samples. The cut-off value of CT was determined using the maximum Youden index. AUC, Area Under Curve. [file 12941_2023_558_MOESM1_ESM.docx]

**Additional File 1**

**CRISPR/Cas12a-based assay for the rapid and** **high-sensitivity detection of *Streptococcus agalactiae* colonization in pregnant women with** **premature rupture of membrane**

Donghong Yu^1,2,3†^, Bin Liang^4†^, Haipo Xu^5,6^, Lu Chen^2,3^, Zhoujie Ye^1,2,3^, Zhihui Wu^7*^ and Xinrui Wang^2,3*^


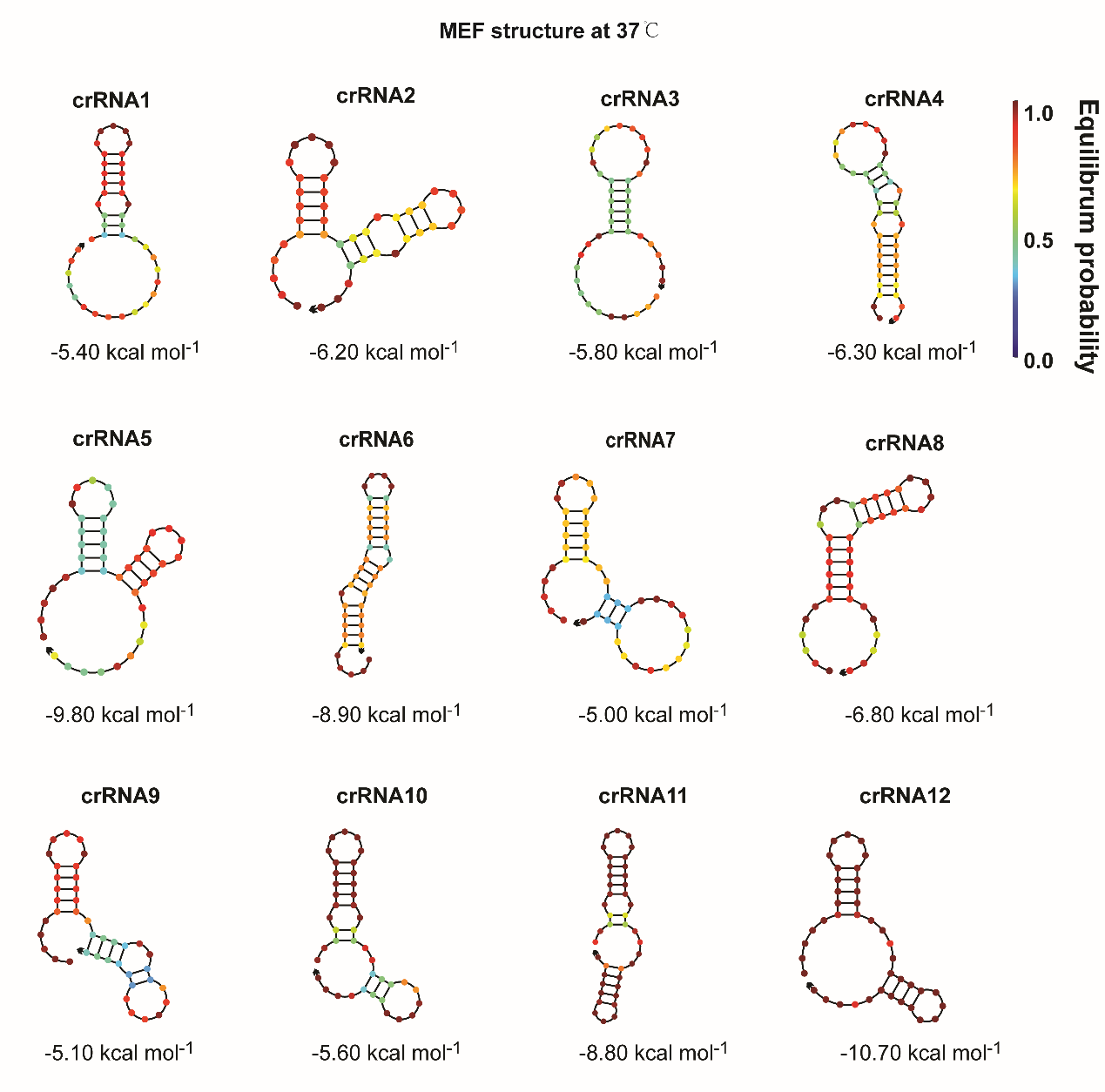


**Fig. S1.** Predicted the second structure and minimum free energy of crRNA1 to crRNA12. *Abbreviation:* MFE, the minimum free energy.


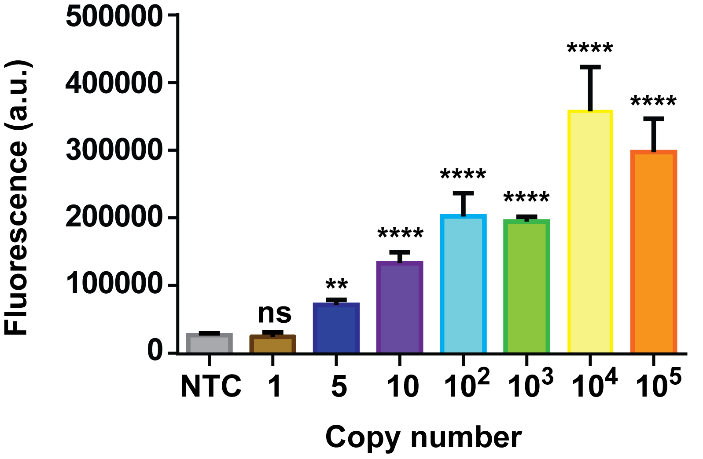


**Fig. S2. Sensitivity analysis of the CRISPR-GBS assay for GBS detection.**

Endpoint fluorescence signals of Cas12a reaction were obtained at 10min. Data represents mean ± SD from octuplicate measurements. Dunnett’s multiple comparisons test was used to analyze the difference from NTC (10^5^, 10^4^, 10^3^, 10^2^, 10^1^ vs NTC). ****P ≤ 0.0001; **P ≤0.05. *Abbreviation*: ns, not significant; NTC, no template control; A.U., arbitrary unit.

**
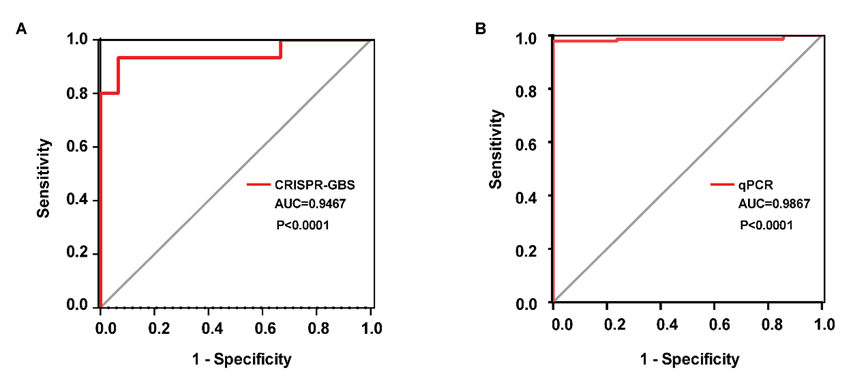
Fig. S3. ROC curve analysis of the CRISPR-GBS assay and qPCR assay on clinical samples.**

**(A)** The ROC analysis of the performance of CRISPR-GBS assay on clinical samples. The cut-off value of CRISPR-GBS assay was determined using the maximum Youden index, which was calculated using the following formula: Youden index = sensitivity + specificity – 1. The ROC curve was statistically analyzed using the GraphPad Prism software (version 5.0), and the Youden Index was determined using Microsoft Excel (2016). **(B)** The ROC analysis of the qPCR assay on clinical samples. The cut-off value of C_T_ was determined using the maximum Youden index. *Abbreviation*: AUC, Area Under Curve.
