# Supplementary material for: CRISPR/Cas12a-based assay for the rapid and high-sensitivity detection of Streptococcus agalactiae colonization in pregnant women with premature rupture of membrane
Source: Ann Clin Microbiol Antimicrob. 2023 Jan 19;22:8. doi: 10.1186/s12941-023-00558-2 (PMC9854146; doi:10.1186/s12941-023-00558-2)
Supplement: Supplementary file 2 — Additional file 2. Table S1. The oligonucleotide sequences of plasmid, primers and probe used in this study. Table S2. The sequences and the corresponding target efficiency score of designed crRNAs. [file 12941_2023_558_MOESM2_ESM.docx]

**Additional File 2**

**CRISPR/Cas12a-based assay for the rapid and high-sensitivity detection of *Streptococcus agalactiae* colonization in pregnant women with premature rupture of membrane**

Donghong Yu^1,2,3†^, Bin Liang^4†^, Haipo Xu^5,6^, Lu Chen^2,3^, Zhoujie Ye^1,2,3^, Zhihui Wu^7*^ and Xinrui Wang^2,3*^

**Table S1.** The oligonucleotide sequences of plasmid, primers and probe used in this study.

| **Name** | **Sequence（5’ to 3’）** |  |  |
| --- | --- | --- | --- |
| **GBS Plasmid**  **(pUC57-*cfb*)** | TATAGGGGGAAAGAAAGCGCTTTGACGACCTTTTGGACAAGTAGTAAGATACCAACATGGGCCCTGTAAATTAAAAATACTGCAGTAGAAGTGATTTTAGTTTAAAGGAGGAAATTTATTATGAACGTTACACATATGATGTATCTATCTGGAACTCTAGTGGCTGGTGCATTGTTATTTTCACCAGCTGTATTAGAAGTACATGCTGATCAAGTGACAACTCCACAAGTGGTAAATCATGTAAATAGTAATAATCAAGCCCAGCAAATGGCTCAAAAGCTTGATCAAGATAGCATTCAGTTGAGAAATATCAAAGATAATGTTCAGGGAACAGATTATGAAAAACCGGTTAATGAGGCTATTACTAGCGTGGAAAAATTAAAGACTTCATTGCGTGCCAACCCTGAGACAGTTTATGATTTGAATTCTATTGGTAGTCGTGTAGAAGCCTTAACAGATGTGATTGAAGCAATCACTTTTTCAACTCAACATTT | |  |
| **RPA primers** |  | **Amplicon Size (bp)** | **Reference** |
| GBS-RPA-F1 | TTTCACCAGCTGTATTAGAAGTACATGCTGATC | 154 | Daher et al. 2014 |
| GBS-RPA-R1* | ACTGTCTCAGGGTTGGCACGCAATGAAGTC |  |  |
| GBS-RPA-F2 | TCTGGAACTCTAGTGGCTGGTGCATTGTTAT | 226 | This study |
| GBS-RPA-R2 | CCACGCTAGTAATAGCCTCATTAACCGGTT |  |  |
| GBS-RPA-F3* | ATGAACGTTACACATATGATGTATCTATCTGGA | 220 | This study |
| GBS-RPA-R3 | CATAATCTGTTCCCTGAACATTATCTTTGAT |  |  |
| **PCR primers** |  |  |  |
| GBS-PCR-F1 | TTTCACCAGCTGTATTAGAAGTA | 154 | Ke et al. 2000 |
| GBS-PCR-R1 | GTTCCCTGAACATTATCTTTGAT |  |  |
| **ssDNA reporter** | 6-FAM-TTATT-BHQ1 |  |  |

*Corresponds to the optimum RPA primers used in this study were labeled with asterisk.

**Table S2.** The sequences and the corresponding target efficiency score of designed crRNAs.

| **Name** | **Sequence (5’-3’)** | **Target efficiency score** |
| --- | --- | --- |
| crRNA1 | UAAUUUCUACUAAGUGUAGAU**GUUUAAAGGAGGAAAUUUAU** | 0.6616 |
| crRNA2 | UAAUUUCUACUAAGUGUAGAU**AAGGAGGAAAUUUAUUAUGA** | 0.7869 |
| crRNA3 | UAAUUUCUACUAAGUGUAGAU**UUAUGAACGUUACACAUAUG** | 0.3007 |
| crRNA4 | UAAUUUCUACUAAGUGUAGAU**ACCAGCUGUAUUAGAAGUAC** | 0.9466 |
| crRNA5 | UAAUUUCUACUAAGUGUAGAU**CCACUUGUGGAGUUGUCACU** | 0.6349 |
| crRNA6 | UAAUUUCUACUAAGUGUAGAU**CAUGAUUUACCACUUGUGGA** | 0.4649 |
| crRNA7 | UAAUUUCUACUAAGUGUAGAU**CUGGGCUUGAUUAUUACUAU** | 0.5856 |
| crRNA8 | UAAUUUCUACUAAGUGUAGAU**AAUUCUAUUGGUAGUCGUGU** | 0.5628 |
| crRNA9* | UAAUUUCUACUAAGUGUAGAU**UCAACUGAAUGCUAUCUUGA** | 0.3801 |
| crRNA10 | UAAUUUCUACUAAGUGUAGAU**AUAUUUCUCAACUGAAUGCU** | 0.3525 |
| crRNA11 | UAAUUUCUACUAAGUGUAGAU**AUAAUCUGUUCCCUGAACAU** | 0.7998 |
| crRNA12 | UAAUUUCUACUAAGUGUAGAU**CACGCUAGUAAUAGCCUCAU** | 0.8641 |

Bold characters represent a crRNA binding region and targeted PAM-proximal truncate sequence.

*The final sequence of crRNA used for CRISPR-GBS assay.

*Abbreviations*: crRNA, guide RNA.
